# Deep learning-based MRI model for predicting P53-mutated hepatocellular carcinoma

**DOI:** 10.1186/s12880-025-02045-w

**Published:** 2025-12-22

**Authors:** Lulu Jia, Qing Yang, Hanchen Jiang, Gang Huang, Zhijun Wang, Xinxin Guo, Jinkui Li, Hao Xu, Junqiang Lei

**Affiliations:** 1 The First Clinical Medical College of Lanzhou University, Lanzhou City, Gansu Province China; 2 Department of Medical Imaging, Anhui Medical University Anqing Medical Center (Anqing Municipal Hospital), Anqing, China; 3 The First Clinical Medical College of Lanzhou University, Lanzhou, China; 4 Department of Statistics, University of California Los Angeles, Los Angeles, CA USA; 5 Department of Radiology, Gansu Provincial Hospital, Lanzhou City, Gansu Province China; 6 Department of Radiology, General Hospital of Ningxia Medical University, Yinchuan City, Ningxia Province China; 7 School of Clinical Medicine, Ningxia Medical University, Yinchuan City, Ningxia Province China; 8 Department of Radiology, The First Hospital of Lanzhou University, No. 1, Donggang West Road, Chengguan District, Gansu Province Lanzhou City, 730000 China; 9 Department of Medicine, Harvard Medical School, Boston, USA; 10 Department of Medicine, Brigham and Women’s Hospital, Boston, USA

**Keywords:** Hepatocellular carcinoma, P53, Deep learning, Magnetic resonance imaging

## Abstract

**Background:**

The P53-mutated Hepatocellular Carcinoma (HCC) is an aggressive variant associated with vascular endothelial growth factor (VEGF) overexpression and increased microvascular density. This study aimed to develop an MRI-based deep learning model for predicting P53-mutated HCC.

**Methods:**

A total of 312 HCC patients who underwent gadolinium-enhanced MRI and were pathologically confirmed between January 2018 and December 2023 were retrospectively enrolled. Participants were randomly divided into training and test dataset at an 8:2 ratio. We developed an EfficientNetV2-based deep learning model, constructing arterial phase (AP) model, portal venous phase (VP), T2-weighted imaging (T2WI), hepatobiliary phase (HBP) single-sequence model, and combined models to predict P53 mutation status. Model performance was evaluated using the area under the curve (AUC), accuracy, sensitivity, specificity, precision, and F1 score as metrics. Differences in AUC values were compared using Delong’s test.

**Results:**

A total of 312 pathologically confirmed HCC patients (age: 56 ± 9 years; male = 240) were included, with a training dataset (*n* = 249) and test dataset (*n* = 63).Among single-sequence models, the HBP model demonstrated superior diagnostic performance (AUC = 0.715) compared to T2WI, AP, and VP models. The multiphase combined model (T2WI + AP + VP) significantly outperformed single-sequence models, achieving AUCs of 0.982 (95% CI: 0.959–1.000) in the training dataset and 0.914 (95% CI: 0.819–1.000) in the test dataset. However, incorporating the HBP sequence into the combined model (T2WI + AP + VP + HBP) did not further improve diagnostic performance (*P* > 0.05).

**Advances in knowledge:**

The combined model incorporating AP, VP, T2WI, and HBP sequences demonstrated numerically highest performance in predicting P53-mutated HCC.

**Supplementary Information:**

The online version contains supplementary material available at 10.1186/s12880-025-02045-w.

## Introduction

Despite significant advances in understanding the molecular mechanisms of hepatocellular carcinoma (HCC), it remains a highly lethal malignancy with limited treatment options [1]. A major barrier to the implementation of effective individualized treatment based on the Barcelona Clinic Liver Cancer stage is the high degree of heterogeneity exhibited by HCC at the genomic, molecular, and histological levels [1, 2].The P53 gene, a well - characterized tumor suppressor, typically functions to induce cell cycle arrest, senescence or apoptosis, and facilitate DNA repair [3]. However, P53 mutations often confer oncogenic properties, which are associated with increased tumor invasiveness, decreased patient survival rates, and a poor response to conventional cancer therapies [4–7]. P53 mutations occur in 12%-48% of HCC cases [8]. At the histological subtype level, P53 mutations are frequently observed in the macrotrabecular-massive (MTM) subtype, a newly defined histological variant of HCC by the World Health Organization in 2019, characterized by its invasive biological behavior and unfavorable prognosis [9, 10]. In light of this, researchers are actively exploring various P53-targeting strategies, including gene therapy, P53-based vaccines, and P53-targeting bispecific antibodies, with the aim of providing personalized treatment options for each patient [11, 12].

Previous studies have demonstrated certain limitations in predicting P53 mutations using conventional imaging features. Although tumor vessel enlargement on CT arterial phase and reduced relative enhancement ratio on gadoxetic acid-enhanced MRI hepatobiliary phase have been significantly associated with P53-mutated HCC [13], the diagnostic specificity remains suboptimal at 63.4%, limiting its clinical utility.Weng et al. revealed significant heterogeneity in imaging manifestations of P53-mutated HCCs across different tumor diameters [14]. Furthermore, Wu’s team employed texture analysis to identify five predictive CT texture parameters (angular second moment [ASM], contrast, correlation, inverse difference moment [IDM], and entropy) for P53 mutation status [15]. However, their study was constrained by a small sample size (*n* = 63), resulting in insufficient statistical power and limited generalizability of findings. Given these methodological constraints, the development of quantitative deep learning-based objective prediction models holds significant clinical importance.

Recent years have witnessed remarkable progress in predicting aggressive HCC subtypes using convolutional neural network (CNN)-based medical image analysis. Liu et al. developed a CNN model that directly extracts deep features from intravoxel incoherent motion diffusion-weighted imaging (IVIM-DWI), achieving successful prediction of microvascular invasion (MVI) with an AUC of 0.81 in the validation cohort [16]. Wang et al. subsequently proposed an end-to-end deep learning framework integrating image preprocessing, automatic segmentation, feature extraction, and MVI prediction modules, which demonstrated outstanding performance (AUC = 0.92) in an independent validation set [17]. Innovatively, Song’s team employed a ResNet-based 2.5D/3D hybrid deep learning architecture to extract latent features from multiphase CT images, combined with a random forest classifier to construct a feature fusion system for accurate prediction of HCC histopathological grading. To our knowledge, no previous studies have investigated deep learning-based differentiation of P53-mutated HCC using multiphase gadoxetic acid-enhanced MRI (EOB-MRI) [18].

Therefore, this study aims to develop a preoperative multiphase MRI-based deep learning model to predict P53-mutated HCC patients undergoing hepatectomy.

## Materials and methods

### Study population

**Fig. 1:**
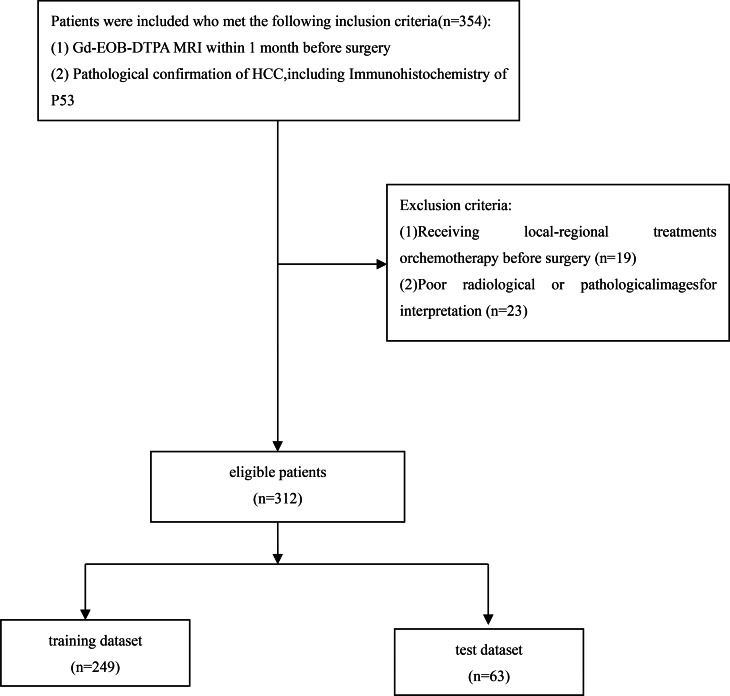
Flowchart of patient inclusion and exclusion in this retrospective study

This retrospective study was conducted in accordance with the ethical guidelines of the Declaration of Helsinki and approved by the Institutional Review Board of the the First Clinical Hospital of Lanzhou University.(LDYYLL-2024-398) Waiver of informed consent was granted due to the retrospective nature of the study. Consecutive patients who underwent surgical resection or liver transplantation for hepatocellular carcinoma (HCC) between January 2018 and December 2023 were enrolled. Inclusion criteria were: (1) Age > 18 years; (2) Preoperative gadolinium-enhanced MRI performed within 1 month before surgery; (3) Pathologically confirmed HCC diagnosis; (4) Available clinical laboratory data and pathological slides; Exclusion criteria: (1) History of locoregional therapy or chemotherapy prior to surgery; (2) Image artifacts or poor image quality (Fig. 1).

### Clinical and laboratory data

Demographic and clinical variables were recorded, including: age, sex, etiology(hepatitis C, hepatitis B, or others), tumor size, alpha-fetoprotein(AFP) level, platelet count, neutrophil count, the neutrophil-to-lymphocyte ratio, prothrombin time, international normalized ratio(INR), aspartate aminotransferase(AST), alanine aminotransferase(ALT), albumin, total bilirubin level and carcinoembryonic antigen(CEA).

### Histologic diagnosis and immunohistochemistry

Histopathological data were retrospectively collected from pathology reports and independently reviewed by two pathologists for all histological and immunohistochemical slides, with recorded parameters including: number of tumor foci, maximum tumor diameter, cirrhosis status in non-tumorous liver tissue, and P53 mutation status; for patients with multifocal lesions, the largest lesion was analyzed as the dominant tumor. The P53 immunohistochemical scoring criteria were: 0 points (negative), 1 point (< 10% positive cells), 2 points (10–50% positive cells), and 3 points (> 50% positive cells), with scores 0–1 defined as non-P53-mutated HCCs and scores 2–3 defined as P53-mutated HCCs.

### MRI examination

MRI scans were performed using GE Medical Systems 3.0T MRI (GE, USA), Philips Medical Systems 1.5T MRI, or Philips Medical Systems 3.0T MRI (Philips Netherlands) imaging system with a 16-channel phased array body coil. The scan ranged from the top to the lower edge of the liver. The images in arterial phase (AP) and portal venous phase (VP) were obtained at 30–35 s and 65–70 s, respectively, after a bolus injection of Gd-EOB-DTPA (Primovist, 0.1 mL/kg body weight) followed by 20 mL saline with a flow rate of 1 mL/s. Hepatobiliary phase (HBP) images were obtained at 20 min after GD-EOB-DTPA administration. The detailed scanning sequences and parameters were shown in Table S1 below.

### Deep learning model construction

**Fig. 2:**
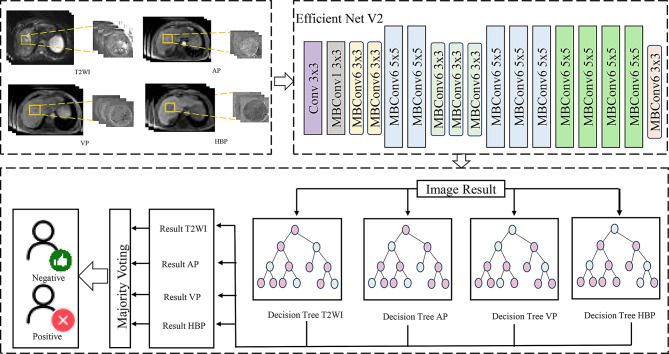
Architecture of the proposed deep learning model.The network employs a hybrid design with EfficientNetV2 backbone for feature extraction from MRI sequences, dynamic depthwise separable convolution blocks (shaded regions), and an attention pooling module compressing features into embedding vectors. Final classification integrates slice-level predictions through a random forest classifier

Developed independent prediction models based on four MRI sequences (arterial phase [AP], portal venous phase [VP], T2-weighted imaging [T2WI], and hepatobiliary phase [HBP]), followed by integrated modeling combining T2WI + AP + VP tri-phase and full-sequence combinations. All imaging data underwent standardized preprocessing including inter-slice registration, intensity normalization (N4 bias field correction), and regions of interest(ROI) cropping. For each phase, 2D regions of interest were manually delineated, and only slices containing ROIs were retained for model training. The EfficientNetV2 [19] architecture (from timm library) was employed as the backbone, featuring dynamic depthwise separable convolution blocks and an attention pooling module that compresses 3D feature maps into discriminative embedding vectors through channel attention mechanisms. For performance optimization, a hybrid loss function was designed combining weighted cross-entropy loss (with class weights inversely proportional to sample size) and label-aware contrastive loss (temperature coefficient τ = 0.7) at an 8:2 ratio. Rigorous data control strategies were implemented during training: reproducibility ensured through fixed random seeds, patient-ID stratified train/test split (8:2 ratio). For single-sequence prediction models, we firstly generated a slice-level probability for each lesion, and then averaged across slices to yield one probability per patient. For tri-phase and full-sequence models, we fused the outputs from each sequence model into a random forest classifier (n_estimators = 100 and maximum tree depth of 5) for the final decision-making. This two-stage architecture effectively integrates local feature extraction capabilities of deep convolutional networks with the global discriminative power of ensemble learning (Fig. 2). The integration of EfficientNetV2 and random forest is motivated by the hypothesis that combining CNN-based feature extraction with tree-based classifiers can mitigate overfitting in small datasets and enhance interpretability. EfficientNetV2 offers scalable and efficient feature representation, while random forests reduce variance and provide interpretable decision-making.

### Statistical analysis

Continuous variables were presented as mean ± standard deviation when normally distributed or otherwise as median and interquartile range (IQR), while categorical variables were displayed as numbers and percentages. Independent samples t-test was used for comparing continuous variables meeting normality and homogeneity of variance assumptions, with Mann-Whitney U test applied for non-normally distributed or heteroscedastic variables. One-way ANOVA or Kruskal-Wallis test was employed to assess differences in continuous variables between training and test datasets, whereas Fisher’s exact test or χ² test was used for categorical variables. Predictive performance was evaluated through receiver operating characteristic (ROC) curve analysis, with area under the curve (AUC) comparisons performed using DeLong’s method.

## Result

### Patient characteristics

Among the 354 initially considered patients, 42 were excluded (Fig. 1), resulting in a final cohort of 312 patients. Among the 312 HCC patients in our cohort, P53 mutations were identified in 83 cases (26.6%).The training dataset comprised 249 patients (80%; mean age 57 ± 9 years [SD]; 194 males [78%], 55 females [22%]), while the test dataset included 63 patients (20%; mean age 55 ± 10 years [SD]; 46 males [73%], 17 females [27%]). Hepatitis B virus infection was the predominant risk factor, present in 249 of 312 patients (80%). When examining laboratory variables as continuous measures, no statistically significant differences were observed between training and test dataset except for ALT levels (*P* = 0.006) (Table 1). Baseline clinical characteristics showed no statistically significant differences between P53-mutated and non-P53-mutated HCC patients in the training dataset (Table 2).

**Table 1:** Clinical and pathologic characteristics of patients with HCC in the training and test data sets

Characteristics	Training Data Set (*n* = 249)	Test Data Set (*n* = 63)	*P* Value
Age(y)*	57 ± 9	55 ± 10	0.222
Sex			0.410
Male	194(78)	46(73)	
Female	55(22)	17(27)	
Etiology			0.542
HBV infection	196(79)	53(84)	
HCV infection	16(6)	4(6)	
Other	37(15)	6(10)	
Serum AFP level^‡^	9.8(3.0-193.0)	4.9(2.0-499.5)	0.284
Platelet count (×10^9^/L)^‡^	124.5(89.8–173.0)	110.0(78.5–169.0)	0.328
Neutrophil count (^9/L) ^‡^	4.1(3.1–5.7)	3.7(2.5–5.5)	0.100
Lymphocyte count (^9/L) ^‡^	1.1(0.8–1.5)	0.9(0.6–1.2)	0.024
The neutrophil-to-lymphocyte ratio^‡^	4.1(2.7–6.1)	4.0(2.7–6.3)	0.906
Prothrombin time (sec)^‡^	13.1(12.1–14.1)	13.1(12.0-14.9)	0.495
INR^‡^	1.2(1.1–1.3)	1.2(1.1–1.4)	0.592
AST level (IU/L)^‡^	46.0(29.8–84.7)	40.0(27.0–64.0)	0.060
ALT level (IU/L)^‡^	69.5(39.5–128.0)	50.5(23.8–95.0)	0.006
ALB level (g/L)^‡^	36.2(32.9–39.0)	35.6(31.9–39.6)	0.471
Total bilirubin level (umol/L)^‡^	21.0(15.8–29.0)	20.6(15.4–28.3)	0.861
CA125^‡^	14.0(8.8–27.2)	16.2(10.6–33.1)	0.068
CEA^‡^	2.0(1.2–3.2)	2.2(1.4–3.5)	0.294
CA199^‡^	13.9(9.6–25.9)	15.0(10.4–27.0)	0.903
MVI	122(49)	31(46)	0.720

Note.-Unless indicated otherwise, data are numbers of patients, with percentages in parentheses. AFP = α-fetoprotein; ALB = albumin; ALT = alanine aminotransferase; AST = aspartate aminotransferase; CEA = Carcino Embryonic Antigen; HCC = hepatocellular carcinoma; HBV = hepatitis B virus; HCV = hepatitis C virus; INR = International normalized ratio; MVI = microvascular invasion

^*^Data are means ± SDs

^‡^Data are medians, with IQRs in parentheses

^ǁ^Statistical significance was found between the training and external test data sets and the internal and external test data sets

**Table 2:** Patient characteristics in the training and test data sets according to the P53 mutations

Characteristics	Training Data Set (*n* = 249)			Test Data Set (*n* = 63)		
	P53-mutated HCC (*n* = 66)	Non-P53-mutated HCC (*n* = 183)	*P* Value	P53-mutated HCC (*n* = 17)	Non-P53-mutated HCC (*n* = 46)	*P* Value
Age(y)*	57 ± 9	56 ± 9	0.465	55 ± 8	55 ± 10	0.888
Sex			0.349			0.048
Male	49(74)	146(80)		9(53)	36(78)	
Female	17(26)	37(20)		8(47)	10(22)	
Etiology			0.583			0.354
HBV infection	49(74)	147(80)		16(92)	37(80)	
HCV infection	5(8)	11(6)		0(0)	4(9)	
Others	12(18)	25(14)		1(6)	5(11)	
Serum AFP level^‡^	16.3(4.0-259.0)	7.9(2.6-190.8)	0.174	4.6(1.4–455.0)	5.1(2.3-507.3)	0.322
Platelet count (×10^9^/L)^‡^	128.0(79.0-155.0)	121.5(92.5-188.5)	0.303	99.0(71.5-162.5)	117(80.5-173.5)	0.441
Neutrophil count (^9/L) ^‡^	3.8(2.8–5.3)	4.3(3.2–6.1)	0.098	3.7(2.0-6.3)	3.7(2.5–5.1)	0.765
Lymphocyte count (^9/L) ‡	1.0(0.7–1.5)	1.1(0.8–1.4)	0.839	1.0(0.6–1.5)	0.9(0.6–1.2)	0.874
The neutrophil-to-lymphocyte ratio^‡^	3.7(2.6–5.8)	4.1(2.7–6.2)	0.323	4.2(2.5–6.8)	4.0(2.7–6.3)	0.908
Prothrombin time (sec)^‡^	12.0(11.3–14.2)	13.1(12.1–14.1)	0.545	13.1(11.7–14.9)	13.1(12.0–15.0)	0.726
INR^‡^	1.2(1.1–1.3)	1.2(1.1–1.3)	0.595	1.2(1.1–1.4)	1.2(1.1–1.4)	0.748
AST level (IU/L)^‡^	47.0(30.0-82.5)	46.0(29.0–86.0)	0.834	36.0(27.0-57.5)	41.0(27.0–68.0)	0.323
ALT level (IU/L)^‡^	72.5(31.0-122.5)	67.5(41.2–128.0)	0.732	38.0(21.5–65.0)	56.0(24.5–104.0)	0.098
ALB level (g/L)^‡^	36.5(32.7–38.4)	36.2(33.1–39.0)	0.740	36.6(34.0-39.8)	34.6(31.8–39.5)	0.268
Total bilirubin level (umol/L)^‡^	23.6(16.3–30.7)	20.9(15.6–27.5)	0.231	21.3(16.6–33.2)	19.8(14.7–27.7)	0.266
CA125^‡^	15.0(11.2–32.4)	13.1(8.0-27.1)	0.086	16.0(10.1–67.0)	16.2(11.0-34.2)	0.853
CEA^‡^	2.2(1.2–3.3)	1.9(1.2–3.2)	0.678	2.1(1.2–3.9)	2.2(1.5–3.4)	0.770
CA199^‡^	14.4(9.2–26.8)	13.8(9.8–25.0)	0.771	13.0(8.9–27.5)	15.7(10.4–27.0)	0.623
MVI	35(53)	87(48)	0.691	8(50)	23(50)	0.813

Note.-Unless indicated otherwise, data are numbers of patients, with percentages in parentheses. AFP = α-fetoprotein, ALB = albumin, ALT = alanine aminotransferase, AST = aspartate aminotransferase, CEA = Carcino - Embryonic Antigen, HBV = hepatitis B virus, HCV = hepatitis C virus, INR = International normalized ratio, MVI = microvascular invasion

^*^Data are means ± SDs

^‡^Data are medians, with IQRs in parentheses

### Diagnostic performance of Single-Phase MRI-Based deep learning models

**Fig. 3:**
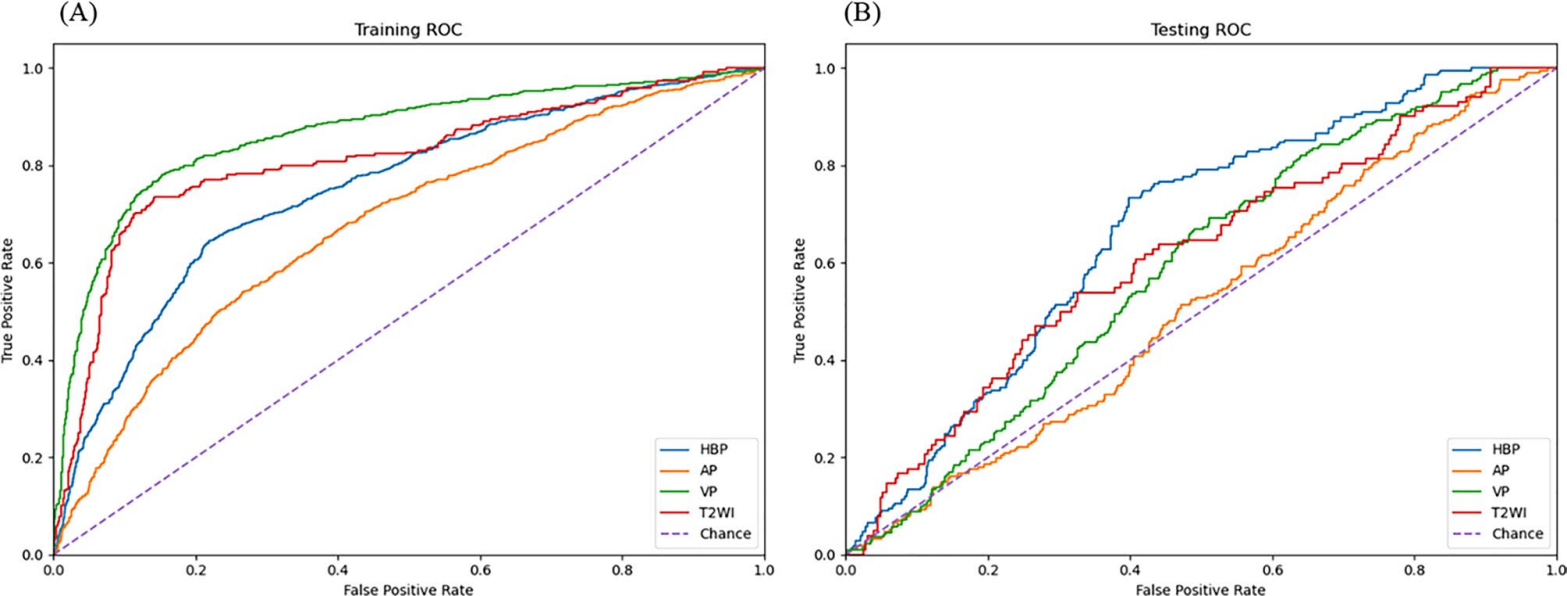
Receiver operating characteristic (ROC) curves of single-phase MRI-based deep learning models for predicting P53-mutated hepatocellular carcinoma. (**A**) Training set: arterial phase (AP, saffron; AUC = 0.93), portal venous phase(VP, green; AUC = 0.74),hepatobiliary phase(HBP, blue; AUC = 0.83) and T2-weighted imaging (T2WI, red; AUC = 0.66) show significant discrimination versus chance line (dashed violet, AUC = 0.50). (**B**) Testing set: Performance remains robust with HBP achieving the highest AUC (0.67; DeLong test *P* < 0.05 for HBP vs. others)

We evaluated our deep learning models on the test dataset using AUC, accuracy, sensitivity, specificity, precision, and F1-score as performance metrics. The T2WI, AP, VP and HBP single-phase deep learning models achieved AUCs of 0.572 (95% CI: 0.507–0.637), 0.544 (95% CI: 0.501–0.588), 0.599 (95% CI: 0.555–0.642), and 0.665 (95% CI: 0.621–0.708) respectively in the test dataset. The ROC curves for these models are shown in Fig. 3. Among these four single-phase models, the HBP model yielded the highest AUC, demonstrating statistically superior performance compared to the T2WI model (*P* = 0.004), AP model (*P* < 0.001), and VP model (*P* = 0.035). Additional diagnostic metrics are presented in Table 3.

### Diagnostic performance of combined deep learning models

**Fig. 4:**
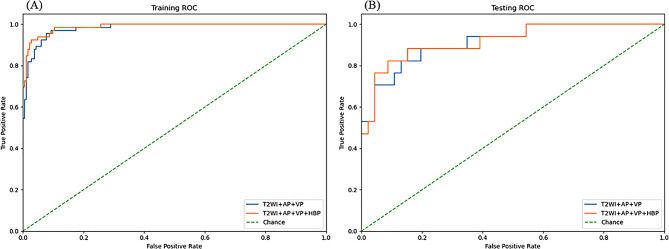
Receiver operating characteristic curves comparing multi-sequence MRI fusion models for P53-mutation prediction in hepatocellular carcinoma. (**A**) Training set performance shows both the tri-phase model (T2WI + AP + VP, blue curve; AUC = 0.98) and full-sequence model (T2WI + AP + VP + HBP, saffron curve; AUC = 0.99) significantly outperform chance level (dashed green line, AUC = 0.5). (**B**) Testing set: While the full-sequence model showed numerically higher AUC (0.92 vs. 0.91 for tri-phase), the difference was not statistically significant (DeLong test, *p* > 0.05)

The tri-phase combined model (T2WI + AP + VP) demonstrated AUCs of 0.982 (95% CI: 0.959-1.000) in the training dataset and 0.914 (95% CI: 0.819-1.000) in the test dataset (Fig. 4). When incorporating the HBP phase to create a quadri-phasemodel (T2WI + AP + VP + HBP), the AUCs improved to 0.988 (95% CI: 0.969-1.000) in training dataset and 0.919 (95% CI: 0.827-1.000) in test dataset. DeLong’s test revealed no statistically significant differences in AUC between the two combined models (training dataset: *P* = 0.157; test dataset: *P* = 0.688). Table 3 presents additional performance metrics including accuracy, precision, sensitivity, and F1-scores.

**Table 3:** Diagnostic performance of deep learning models for predicting P53 mutations

Model	Datasets	AUC (95% CI)	Accuracy	Precision	Sensitivity	Specificity	F1-Score
T2WI	Training Data Set	0.664(0.625,0.702)	0.660	0.635	0.608	0.681	0.625
	Test Data Set	0.572(0.507,0.637)	0.704	0.486	0.098	0.891	0.478
AP	Training Data Set	0.932(0.919,0.945)	0.885	0.876	0.885	0.885	0.867
	Test Data Set	0.544(0.501,0.588)	0.624	0.514	0.338	0.699	0.510
VP	Training Data Set	0.744(0.721,0.767)	0.750	0.714	0.629	0.803	0.712
	Test Data Set	0.599(0.555,0.642)	0.574	0.539	0.531	0.586	0.520
HBP	Training Data Set	0.833(0.814,0.852)	0.800	0.762	0.674	0.852	0.761
	Test Data Set	0.665(0.621,0.708)	0.620	0.606	0.676	0.604	0.575
T2WI + AP + VP	Training Data Set	0.982(0.959,1.000)	0.924	0.961	0.742	0.989	0.838
	Test Data Set	0.914(0.819,1.000)	0.857	0.750	0.706	0.913	0.727
T2WI + AP + VP + HBP	Training Data Set	0.988(0.969,1.000)	0.932	0.962	0.773	0.989	0.857
	Test Data Set	0.919(0.827,1.000)	0.889	0.813	0.765	0.935	0.788

Note- AUC = arca under the receiver operatingcharacteristic curve; T2WI = T2-weighted imaging; AP = arterial phase; VP = portal venous phase; HBP = hepatobiliary phase

### Comparative diagnostic performance of combined versus Single-Phase MRI deep learning models

In the test dataset, the quadri-phaseT2WI + AP + VP + HBP model demonstrated significantly superior AUC compared to all single-phase models (all *P* < 0.001). Notably, it achieved a 38.2% AUC improvement over the best-performing single-phase HBP model (0.919 vs. 0.665). The combined model’s 95% CI lower bound (0.827) exceeded the upper bounds of all single-phase models, indicating that multi-sequence integration substantially overcomes the performance limitations of single-phase approaches (AUC improvement range: 38.2%-68.9%). The quadri-phaseanalysis also showed markedly enhanced predictive stability, with CI widths 35%-50% narrower than single-phase models.

Similarly, the tri-phase T2WI + AP + VP model outperformed all single-phase models in the test dataset (all *P* < 0.001), delivering a 37.4% AUC increase versus the top single-phase HBP model (0.914 vs. 0.665). Its 95% CI lower limit (0.819) surpassed all single-phase models’ upper bounds, demonstrating that multi-sequence fusion effectively breaks through the performance ceiling of individual sequences (elevating AUC from 0.544 to 0.665 to 0.914).

## Discussion

The P53-mutated hepatocellular carcinoma (HCC), as an aggressive subtype with distinct clinicopathological characteristics, holds significant clinical value for precise preoperative identification to guide individualized treatment strategies. This study innovatively developed a deep learning model based on multiparametric contrast-enhanced MRI to evaluate the diagnostic performance of individual MRI sequences and their combinations for predicting P53 mutation status. Our findings revealed that among single-sequence analyses, the hepatobiliary phase (HBP) demonstrated relatively optimal predictive performance (AUC = 0.665, 95% CI: 0.621–0.708). The multi-sequence fusion models showed statistically significant improvement, with the tri-phase T2WI + AP + VP combination achieving excellent predictive accuracy (AUC = 0.914, 95% CI: 0.819-1.000). Notably, although the quadri-phaseT2WI + AP + VP + HBP model showed marginal AUC improvement (0.919, 95% CI: 0.827-1.000), DeLong’s test indicated this difference was not statistically significant (*P* = 0.688).

Serum alpha-fetoprotein (AFP) levels have been established as an independent predictor for aggressive HCC subtypes [20–25], including those with P53 mutations, and are associated with tumor biological behavior [13]. However, our study revealed no significant difference in AFP levels between P53-mutated and wild-type HCC cases. This discrepancy may be attributed to several factors: (a)The biological effects of AFP in P53-mutated subgroups might be masked by concurrent driver mutations (e.g., β-catenin mutations) [26]; (b) Conventional AFP cutoff values (e.g., 400 ng/mL) demonstrate insufficient sensitivity for P53-specific subtyping; and (c) Our cohort predominantly consisted of hepatitis B virus-associated HCC (80%), whereas previous studies mainly focused on hepatitis C-related cases - this etiological difference may lead to distinct AFP expression profiles. Importantly, the binary classification of P53 status (P53-mutated HCC vs. non-P53-mutated HCC) may obscure deeper biological correlations. For instance, the aflatoxin-associated R249S hotspot mutation may exhibit unique AFP expression patterns not shared by other P53 mutation types. These findings suggest that relying solely on AFP levels for P53 status determination in clinical practice could lead to misclassification [27]. We therefore recommend integrated assessment combining deep learning/radiomic features or circulating tumor DNA analysis for more accurate stratification.

Among the four single-sequence models, the hepatobiliary phase (HBP) model demonstrated superior performance in predicting P53 mutation status, achieving AUCs of 0.833 (training dataset) and 0.665 (test dataset). This finding aligns with established literature [13] showing that P53-mutated HCC exhibits characteristic reductions in the relative enhancement ratio (RER) during HBP imaging—a phenomenon mechanistically linked to downregulated expression of the OATP1B3 transporter on tumor cell membranes [28]. At the molecular level, P53 mutation likely suppresses OATP1B3 through two synergistic pathways: Genetic mutual exclusivity: P53 mutation and Wnt/β-catenin pathway activation (a key positive regulator of OATP1B3) are rarely co-occurring in HCC [29]. Transcriptional repression: Mutant P53 downregulates hepatocyte nuclear factor 4-α (HNF4α), a master transcriptional activator of OATP1B3 gene expression [30]. Our deep learning approach provides the first in vivo validation of this biology-imaging correlation by extracting subvisual features from HBP sequences. The model-identified imaging signatures likely reflect aberrant contrast agent kinetics secondary to OATP1B3 dysfunction in mutated tumors.

Although the HBP single-sequence model demonstrated relatively optimal diagnostic performance, its incorporation into the T2WI + AP + VP combined model did not yield statistically significant improvement in diagnostic efficacy (ΔAUC = 0.005, *P* = 0.688), consistent with previous studies predicting HCC microvascular invasion (MVI) [31]. From a feature complementarity perspective, the tri-phase combination (T2WI + AP + VP) likely already captures the major imaging biomarkers of P53 mutation: T2-weighted imaging (T2WI) reflects tumor cellularity and water content, while the arterial phase captures characteristics of tumor neovascularization, and the portal venous phase demonstrates fibrotic features of the tumoral capsule [31–35], collectively containing most diagnostic information needed for P53 mutation detection, rendering HBP’s additional hepatobiliary metabolic data of limited incremental value. Furthermore, From a model architecture perspective, when the baseline model (triple-sequence) has already achieved high diagnostic performance (AUC = 0.914), the marginal benefit of any additional features would naturally diminish. This phenomenon is recognized in machine learning as the “performance saturation effect” [36]. This is particularly evident with limited sample sizes (n = 63 in this study) where models show reduced sensitivity to subtle feature variations. Clinically, these findings are significant: the tri-phase protocol (T2WI + AP + VP) maintains diagnostic performance while reducing scan time by approximately 15–20 minutes and lowering costs, substantially optimizing workflow efficiency, though HBP may retain diagnostic value for specific subpopulations (e.g., small HCCs or atypically enhancing lesions), warranting further investigation.

Deep learning methods have demonstrated superior performance over radiomics features in numerous tasks including lesion detection, prognosis prediction, and multimodal image registration [37–40], with image-based deep learning techniques being widely applied in HCC for characterizing biological features, treatment response, and prognosis [41–43]. Dong et al. [44] developed a multi-sequence radiomic deep learning approach for predicting vessels encapsulating tumor cluster (VETC) with an AUC of 0.844, while Chen et al. [45] compared four different machine learning methods and found that deep learning with clinical features achieved the best performance (AUC = 0.97) in predicting treatment response for HCC patients undergoing transarterial chemoembolization(TACE). Building upon these successful applications of deep learning in HCC patients, we explored the multi-phase MRI fusion network (MPF-Net) model which typically enables more robust and automated image analysis without requiring export intervention. Furthermore, we exclusively used 2D ROIs rather than whole-slide images for deep learning feature extraction, which yielded strong performance consistent with previous studies. This ROI-based approach allowed the model to focus on the most relevant tumor regions, capturing detailed local features that reflect tumor size, shape, and heterogeneity. Our deep learning model employs a multi-input network architecture, and since we utilized four image sequences for P53-mutated hepatocellular carcinoma, we first evaluated the effectiveness of individual modalities before assessing multimodal combinations, which confirmed that single modalities underperformed compared to multimodal approaches.

This study has a few limitations. First, this is a retrospective study with limited data size, underscoring the need for larger, multicenter studies to confirm the generalizability and robustness of the findings. Second, 80% of the participants had chronic hepatitis B as the risk factor for HCC, which may constrain the applicability of the results to populations with differing etiological profiles. Lastly, only four sequences (T2WI, AP, PVP, and HBP) were utilized, and we believe incorporating additional imaging sequences such as T1-weighted imaging (T1WI), and apparent diffusion coefficient(ADC) could potentially improve our model’s performance [46, 47].

## Conclusions

In summary, we have successfully developed an MRI-based deep learning model for predicting P53-mutated hepatocellular carcinoma.

## Supplementary Information

Below is the link to the electronic supplementary material.

Supplementary Material 1

## Data Availability

The datasets used and analysed during the current study are available from the corresponding author on reasonable request.

## Abbreviations

HCC: Hepatocellular carcinoma
VEGF: Vascular endothelial growth factor
AP: A rterial phase
VP: Portal venous phase
T2WI: T2-weighted imaging
HBP: Hepatobiliary phase
AUC: Area under the curve
CT: Computed tomography
MRI: Magnetic resonance imaging
CNNs: Convolutional neural networks
AFP: Alpha-fetoprotein
INR: Nternational normalized ratio
AST: Aspartate aminotransferase
ALT: Alanine aminotransferase
CEA: Carcinoembryonic antigen
RER: Relative enhancement ratio
