# Supplementary material for: Deep learning-based MRI model for predicting P53-mutated hepatocellular carcinoma
Source: BMC Med Imaging. 2025 Dec 22;25:506. doi: 10.1186/s12880-025-02045-w (PMC12723874; doi:10.1186/s12880-025-02045-w)
Supplement: Supplementary file 1 — Supplementary Material 1 [file 12880_2025_2045_MOESM1_ESM.docx]

Table S1

The MRI Scan Sequences and Parameters for Three Medical Systems

| Sequence | Category | TR (ms) | TE (ms) | FOV  (mm) | Thickness  (mm) | Fat Saturation | Breath-hold |
| --- | --- | --- | --- | --- | --- | --- | --- |
| **GE Medical Systems** |  |  |  |  |  |  |  |
| T1WI in/opposed phase | Fast SPGR | 4.6 | 2.0 | 400×400 | 3.0 | No | Yes |
| T1WI-fs | LAVA-Flex | 4.6 | 2.0 | 400×400 | 3.0 | Yes | Yes |
| T2WI-fs | FRFSE-XL | 2857 | 81 | 400×400 | 6.5 | Yes | Yes |
| DWI | DW EPI | 3333 | 68 | 400×400 | 6.5 | Yes | No |
| Contrast-enhanced |  |  |  |  |  |  |  |
| T1WI-fs (AP) | LAVA-Flex | 3.7 | 1.4 | 400×320 | 3.0 | Yes | Yes |
| T1WI-fs (VP) | LAVA-Flex | 3.7 | 1.4 | 400×320 | 3.0 | Yes | Yes |
| T1WI-fs (HBP) | LAVA-Flex | 3.7 | 1.4 | 400×320 | 3.0 | Yes | Yes |
| **Philips Medical Systems**  **1.5T** |  |  |  |  |  |  |  |
| T1WI in/opposed phase | mDIXON | 5.5 | 1.7 | 400×300 | 3.0 | No | Yes |
| T1WI-fs | mDIXON | 5.5 | 1.7 | 400×300 | 3.0 | Yes | Yes |
| T2WI-fs | TSE | 2438 | 100 | 380×380 | 6.6 |  |  |
| DWI | DW EPI | 1264 | 78 | 400×313 | 6.6 | Yes | No |
| Contrast-enhanced |  |  |  |  |  |  |  |
| T1WI-fs (AP) | mDIXON | 5.6 | 1.75 | 375×348 | 3.0 | Yes | Yes |
| T1WI-fs (VP) | mDIXON | 5.6 | 1.75 | 375×348 | 3.0 | Yes | Yes |
| T1WI-fs (HBP) | mDIXON | 5.6 | 1.75 | 375×348 | 3.0 | Yes | Yes |
| **Philips Medical Systems 3.0T** |  |  |  |  |  |  |  |
| T1WI in/opposed phase | mDIXON | 3.1 | 1.09 | 400×350 | 3.0 | No | Yes |
| T1WI-fs | mDIXON | 3.1 | 1.09 | 400×350 | 3.0 | Yes | Yes |
| T2WI-fs | TSE | 2000 | 92 | 360×360 | 6.6 |  |  |
| DWI | DW EPI | 1547 | 55 | 350×420 | 6.6 | Yes | No |
| Contrast-enhanced |  |  |  |  |  |  | Yes |
| T1WI-fs (AP) | mDIXON | 3.7 | 1.31 | 450×322 | 3.0 | Yes | Yes |
| T1WI-fs (VP) | mDIXON | 3.7 | 1.31 | 450×322 | 3.0 | Yes | Yes |
| T1WI-fs (HBP) | mDIXON | 3.7 | 1.31 | 450×322 | 3.0 | Yes | Yes |

Note.—FOV = field of view, fs = fat suppression, AP = arterial phase, VP = portal venous phase, HBP = hepatobiliary phase, Fast SPGR = Fast Spoiled Gradient Recalled Echo, LAVA-Flex = Liver Acquisition with Volume Acceleration-Flexible, FRFSE-XL = Fast Recovery Fast Spin Echo-eXtended Echo Train Length, DW EPI = Diffusion-Weighted Echo Planar Imaging, mDIXON = modified Dixon, TSE = turbo spin-echo.
